# Relationship Between Traffic Volume and Accident Frequency at Intersections

**DOI:** 10.3390/ijerph17041393

**Published:** 2020-02-21

**Authors:** Angus Eugene Retallack, Bertram Ostendorf

**Affiliations:** Faculty of Sciences, School of Biological Sciences, The University of Adelaide, North Terrace Campus, Adelaide, SA 5005, Australia; angus.retallack@adelaide.edu.au

**Keywords:** traffic volume, congestion, intersections, rainfall risk, relative risk, urban

## Abstract

Driven by the high social costs and emotional trauma that result from traffic accidents around the world, research into understanding the factors that influence accident occurrence is critical. There is a lack of consensus about how the management of congestion may affect traffic accidents. This paper aims to improve our understanding of this relationship by analysing accidents at 120 intersections in Adelaide, Australia. Data comprised of 1629 motor vehicle accidents with traffic volumes from a dataset of more than five million hourly measurements. The effect of rainfall was also examined. Results showed an approximately linear relationship between traffic volume and accident frequency at lower traffic volumes. In the highest traffic volumes, poisson and negative binomial models showed a significant quadratic explanatory term as accident frequency increases at a higher rate. This implies that focusing management efforts on avoiding these conditions would be most effective in reducing accident frequency. The relative risk of rainfall on accident frequency decreases with increasing congestion index. Accident risk is five times greater during rain at low congestion levels, successively decreasing to no elevated risk at the highest congestion level. No significant effect of congestion index on accident severity was detected.

## 1. Introduction

Traffic accidents were estimated to cost Australia A$33.15 billion in 2016 [1]. This figure comes from estimations of the “value of a statistical life” [2] and costs associated with loss of economic output as a result of injury as well as the repair of property [3]. In the United States, fatalities from traffic accidents surpassed the combined toll taken by the two most deadly diseases, cancer and heart disease, and close to half of the deaths of 19-year-olds were a result of traffic accidents [4]. While annual road fatalities per 100,000 people in Australia were five times less in 2013 compared to 1975 [5], there were over 1100 fatalities in 2018 [6]. To maintain this reduction in fatalities and reduce accidents overall, understanding the range of causative factors that influence traffic accidents is critical.

Influencing factors include environmental conditions [7,8,9,10,11,12,13], vehicle factors [4,14], driver characteristics and behaviour [15,16,17,18], and road design [19,20].

Of particular interest is the effect of traffic conditions on accidents. While on the surface it seems desirable to reduce congestion, if it correlates negatively with serious injury or fatal accident frequency, a reduction may negatively affect road safety [21]. A strong understanding of this relationship is necessary to improve traffic management and reduce accident frequency. Research stems from the 1930s [11], with relationships between accident occurrence and traffic volume/congestion falling into one of two broad categories: linear and non-linear [22].

Veh [11] found a positive correlation between accident rates and average daily traffic (ADT), before accident rates gradually declined in higher traffic volumes; a trend also found by Raff [23]. Other studies using ADT and annual average daily traffic (AADT) reported simple positive linear correlations [24,25,26,27,28]. Gwynn [29] suggested that the higher temporal resolution of hourly traffic data may give a stronger relationship. Using hourly volumes, both Gwynn [29] and Ceder [30] found a U-shaped curve, with the highest accident rates existing in the lowest and highest traffic volumes. Martin [31] also found a U-shaped response, as did Frantzeskakis and Iordanis [32] when considering the relationship to level of service. Shefer [33] hypothesized that the relationship between the volume/capacity (v/c) ratio and fatal accident frequency would form a bell-shaped curve. This hypothesis was supported by Martin [31] when looking at overall accident frequencies (not only fatal) and hourly traffic when using 6-minute traffic volume measurements for periods when accidents occurred.

Fortunately, accidents are rare events. However, from a statistical perspective, this requires analysis over long time periods and broad spatial scales to ensure sufficient sample sizes. Large datasets improve the sensitivity of model response to variables of interest. Advanced modelling approaches use high temporal resolution traffic volume data in combination with multiple covariates to predict accident frequencies, as in a study by Theofilatos [34]. But these approaches cannot be easily adopted elsewhere if the data required to include these covariates is not available (e.g., road geometry, moisture conditions or light levels) and more parsimonious models may be necessary [35]. It is important to note that a parsimonious approach could lead to issues relating to unobserved heterogeneity in unincluded factors [35] between different accidents and intersections. Approaches such as the v/c ratio exist to standardise traffic volume with relation to intersection capacity [36] and allow detailed analysis at a limited number of locations by taking differences in intersection characteristics into account. But the application of this method across broad spatial scales is difficult if road geometry, directional traffic volume, or traffic signal data is unavailable.

This study aims to analyse how traffic volumes affect accident frequency to address the lack of consensus between the linear and non-linear hypotheses in the wake of past research. Large datasets of high temporal frequency traffic volumes are used and the response of accident occurrence to congestion across 120 intersections will be analysed. Separate analyses look at the effect of congestion on accident severity and the effects of rainfall on accident risk across these congestion levels. The City of Adelaide is chosen due to overlap in availability of high temporal frequency, spatially explicit traffic data and detailed accident records.

## 2. Materials and Methods

This analysis combines detailed spatio-temporal traffic accident records and hourly intersection traffic volume data. By normalising traffic volumes to each intersection, the resulting congestion index allowed traffic conditions to be compared between intersections irrespective of differences in intersection characteristics.

Two further factors were investigated. Accidents risks at different congestion levels were analysed with relation to rainfall and accidents were disaggregated by severity level to uncover any influence of congestion on accident severity.

Data was processed and analysed using the R programming language [37] with the RStudio integrated development environment [38].

### 2.1. Study Area

**Figure 1:**
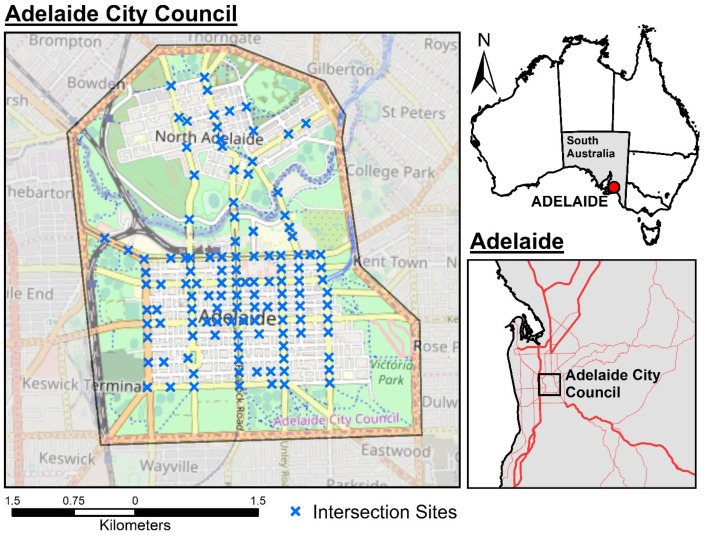
Location of the study area and intersection sites where high temporal resolution traffic volume data exists.

The study is constrained to the Adelaide City Council (ACC) area in South Australia, Australia—chosen based on the extent of the hourly intersection traffic volumes dataset. Figure 1 shows the location relative to wider Adelaide and Australia as a whole.

### 2.2. Data Processing Workflow

**Figure 2:**
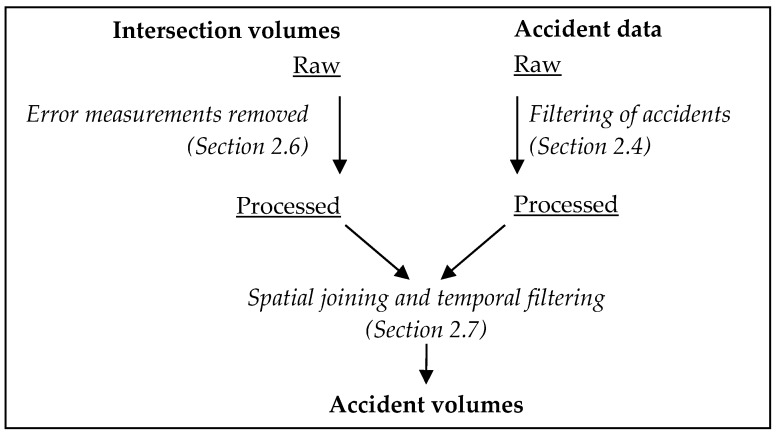
Workflow for processing and joining the intersection traffic volumes and traffic accident datasets.

Figure 2 summarises the process by which intersection traffic volumes were joined to traffic accident records with reference to the relevant methods sections.

### 2.3. Accident Data

Traffic accident records were obtained from the Department of Planning, Transport and Infrastructure’s (DPTI) “Road Crash Data” dataset [39]. While this is publicly available, the dates and times of individual accidents are omitted for privacy reasons. The DPTI provided a version with dates and times included for use in this research. The dataset contains information about each accident, including the date and time, coordinates, weather conditions and accident severity. 

A separate “units” table provides additional information about the units (including cars, cyclists and fixed objects) involved in each accident.

### 2.4. Processing Accident Data

Accidents that included unit types such as cyclists, pedestrians, wheelchairs and animals were removed as these units are not affected by traffic in the same way as vehicles in the main traffic stream.

The date-times of each accident were formatted into the ISO 8610 date-time format with the Adelaide time-zone specified. Standardising date-times between datasets will ensure the accurate temporal joining of accidents and traffic volume measurements. 

As traffic volume data only exists for intersections within the ACC between the years 2010 and 2014, the accidents were filtered to fit these parameters, leaving 2336 accidents (Table 1). Accident times were rounded down to the nearest hour to match the hourly timestamps of the traffic volume data. It was essential to round times down to the previous hour to ensure the traffic volume used was not affected by the accident itself. This practice is used in previous studies looking at the relationship between traffic volume and accident frequencies [34,40,41].

### 2.5. Intersection Traffic Volume Data

Traffic intersection volumes from 2010 to 2014 [42] are publicly available through data.sa.gov.au. The dataset consists of hourly traffic volume measurements for 122 intersections in the ACC; recorded using the Sydney Coordinated Adaptive Traffic System (SCATS). Traffic volumes represent the total number of vehicles to pass through an intersection in each hour. Directional traffic data was not available—subsequent methods detail the approach used to address this. Each hourly measurement includes the coordinates of its corresponding intersection, meaning that every measurement at each intersection has a separate spatial data point. Over five million hourly traffic volume measurements are available to be paired to individual accidents (Table 2). This is important due to the rarity of accident events. A large traffic volume dataset increases the probability that any individual accident will have associated traffic data and increases the number of accidents useable in the analysis.

### 2.6. Processing Intersection Traffic Volume Data

Upon investigation of the data, an intersection on Anzac Highway and one on Wakefield Street were found to have median traffic volumes of zero vehicles per hour. Just over half of the traffic volume measurements at the Wakefield street site were zero and nearly all measurements at the Anzac Highway site were zero; this is unrealistic for two major roads in the ACC. Volume measurements from these two intersections were removed from the dataset, leaving a total of 120 intersections (Table 2). There were large groups of consecutive zero vehicles per hour readings—often during hours of the day where volumes above zero would be expected—these are also errors. To address this, groups of traffic volume measurements that remained the same for more than five consecutive hourly periods, including values above zero, were removed. Overall, removing error measurements reduced the number of traffic volume measurements by approximately 150,000 (Table 2).

Hour of day and date columns were combined into one date-time column and formatted in the ISO 8601 date-time format [43] with the Adelaide time-zone specified.

Traffic volume measurements were also corrected using the provided error ratio, which indicates the proportion of vehicle counts in each hourly period that were made in error. The inverse of this ratio is the “valid ratio”; the proportion of vehicles that were counted correctly in any given hour. Each hourly traffic volume measurement was multiplied by its valid ratio to give a corrected measurement, accounting for error in the SCATS sensors. The SCATS system is developed by the New South Wales Government in Australia and uses data collected from detectors at intersections to manage traffic signals.

### 2.7. Joining Accident and Traffic Volume Datasets

Before analysis of the effects of traffic volumes on the frequency of accidents could be conducted, it was necessary to know the volume of traffic passing through an intersection immediately before each accident. This required joining the accident and intersection traffic volume datasets.

Using the coordinates of each accident and each traffic volume measurement, the two datasets were spatially joined with a distance parameter of 20 m; joining each accident to the traffic volume data for any intersection within 20 m. Because every hourly traffic volume measurement has its own spatial data point, each accident record was duplicated across every traffic volume measurement at its intersection. This large dataset was filtered to only include rows where the date-time of the accident matched with the date-time of the traffic volume measurement. This resulted in a total of 1629 accidents (Table 3) with associated traffic volumes. This new table will be referred to as the accident volumes dataset.

The accidents in this dataset were then used to analyse the effects of traffic volumes on the occurrence of accidents.

### 2.8. Rainfall Data

High temporal resolution rainfall data was purchased from the Bureau of Meteorology (BOM) [44]. Data for the “Adelaide (Kent Town)” rainfall station was available from 1995 to 2015, with a total of 353,439 measurements. Rainfall rates were taken in increments of 0.2 mm with a temporal resolution of 30 minutes.

### 2.9. Accounting for Variability in Intersection Capacity

As each intersection has a different capacity, traffic volumes are not comparable between them. For example, 1000 vehicles per hour may be close to the capacity of one intersection but easily within the capacity of another.

To account for this, traffic volumes must be normalised. Traditionally, v/c ratios are used, with methods derived from the Highway Capacity Manual [36]. If this method were to be used, the capacity at signalized intersections would be calculated individually for different lane groups using their capacity, adjusted saturation flow rate, effective green traffic signal ratio and cycle length [36]. However, the broad spatial scale of the study area makes the use of this method difficult. Signal timing data for each intersection was not easily accessible and intersection geometry information would have been difficult to ascertain and use over 120 intersections. Additionally, traffic volume data was only available as a total count for each intersection. The lack of directional vehicle counts makes the calculation of v/c ratios for different lane groups impossible. As a result, a novel approach to standardising traffic conditions was taken.

This was achieved by assigning each measurement into one of 15 bins in a quantile classification based on other measurements at the same intersection (Appendix A explains the choice of 15 bins). A traffic volume measurement of 300 vehicles per hour may be assigned to bin 15 at a low-volume intersection, while a measurement of 5000 vehicles per hour may be assigned to bin 15 at a high-volume intersection. Looking at the two traffic volumes alone, they seem incomparable; however, they both fall among the highest volume measurements for their respective intersections. These bins effectively act as an index for congestion by representing traffic volumes relative to the overall range of volumes at an intersection.

### 2.10. Analysing the Relationship Between Traffic Volume and Accident Frequency

Intersections were grouped into three different sizes based on their median traffic volumes. Accidents in the accident volumes dataset were then grouped by the size of the intersection they occurred at and the congestion level at the time of the accident. This results in 45 groups (three intersection size ranks × 15 congestion levels). The number that occurred in each of these 45 groups was counted.

However, plotting accident frequencies against congestion index on a linear scale results in a transformation of the response of accident frequency. This is because the 15 congestion levels are not distributed evenly throughout the traffic volume distributions at intersections. To combat this, the median traffic volumes of each of the congestion levels was calculated, as explained in Appendix B.

Accident frequencies were then able to be plotted against this median value, allowing the linear hypothesis to be tested. 

To determine whether the response of accident frequency to traffic volume was linear, or whether a significant non-linear effect was present, various models were fitted to accident frequency for each intersection size. As the data is non-negative count data, regular linear models are not appropriate.

Initially, poisson generalized linear models (GLM) were fit with a single linear explanatory term. These models were then tested for overdispersion to determine whether the poisson was appropriate. If the poisson model is overdispersed, the negative binomial model is more appropriate. The following formulae were used for either the poisson or negative binomial models.

**Linear:**  accident frequency ~ traffic volume
**Quadratic:**  accident frequency ~ traffic volume + (traffic volume)^2^
**Natural** **Spline:** accident frequency ~ natural spline (traffic volume, 4 d.f.)

The most preferable of these three models for each intersection rank were determined using the AICc (Akaike Information Criterion) model selection criterion [45].

### 2.11. Accident Severity Analysis

For analysis of how congestion affects accident severity, the accident volumes dataset was filtered into three subsets, containing property damage only (PDO), minor injury (MI) and serious injury (SI) accidents (there were no fatal accidents at intersections in the ACC during the study period). As there were only 20 SI accidents with traffic volume data, there was too much noise for a clear response to be observed and SI accidents were not considered further.

The frequency of PDO and MI traffic accidents in each congestion level were then plotted, allowing any difference in response of accident frequency to be seen. Normalised frequencies are the proportion of total PDO or MI accident counts in each congestion level. The accident frequency ratio is the ratio of PDO to MI frequencies in each congestion level.

### 2.12. Rainfall Risk Analysis

To determine the effect of rain on accident occurrence, the accidents in the accident volumes table were separated into accidents occurring while it was raining and while it was not raining. For each of these filtered datasets, the accident frequency in each congestion level was counted.

Using these not-raining and raining accident frequencies, the risk of not-raining and raining accidents in each congestion level were calculated. Accident risk is the probability of an accident occurring within a period. Using raining accident risk as an example, risk was calculated using Equation (1):

Raining accident risk = Total # of accidents while raining/Total # of periods in which it was raining,(1)

where a period refers to the hourly traffic volume periods.

To understand how rainfall risk changes with increasing congestion, this calculation was applied to each of the 15 congestion levels (Equation (2)).

Raining accident risk (Cx) = Total # of Cx accidents while raining/Total # of Cx periods in which it was raining,(2)

where Cx is the congestion level (1–15).

The process was repeated for not-raining accidents.

To determine the total number of hourly periods in which it was raining (or not raining), the BOM rainfall data was joined to the intersection volumes table. The number of traffic volume measurement periods in which it was raining and not raining were counted for each of the 15 levels of congestion, allowing risks to be calculated for each level.

Relative risk (RR) is the ratio of the risk of an event occurring under exposed conditions to the risk of an event occurring under control conditions [46]. In the context of accident risks, RR was calculated for each congestion level (Cx) using the following equation and the risk values calculated using Equations (1) and (2).

Relative risk (Cx) = Raining Accident Risk (Cx) / Not-Raining Accident Risk (Cx),(3)

RR was then plotted against congestion index to allow any changes with increasing congestion to be observed. A change in RR would indicate a change in how rainfall affects the risk of an accident. A RR of greater than one means that the risk of an accident occurring is higher when it is raining, while a RR of less than one means that the risk of an accident occurring is greater while it is not raining.

## 3. Results

### 3.1. Relationship Between Traffic Volume and Accident Frequency

Based on Table 4, poisson GLMs were appropriate for the counts of accidents occurring in low-volume intersections. As the poisson is overdispersed for middle- and high-volume intersections, negative binomial models were used for these instead.

Considering the rule of thumb that a delta AIC of two or more gives substantial support for the highest-ranked model [47], the quadratic models are favourable for accident counts at low- and middle-volume intersections, with evidence ratios of 17.7 and 11.5, respectively (Table 5). The delta AICc between the quadratic and natural spline negative binomial models was only 1.2 for high-volume intersections (Table 5), and so the AICc values do not support the choice of one model over the other. However, a delta AICc of 7.8 for the model using only a linear explanatory term indicates a significantly better fit of the two non-linear models.

**Figure 3:**
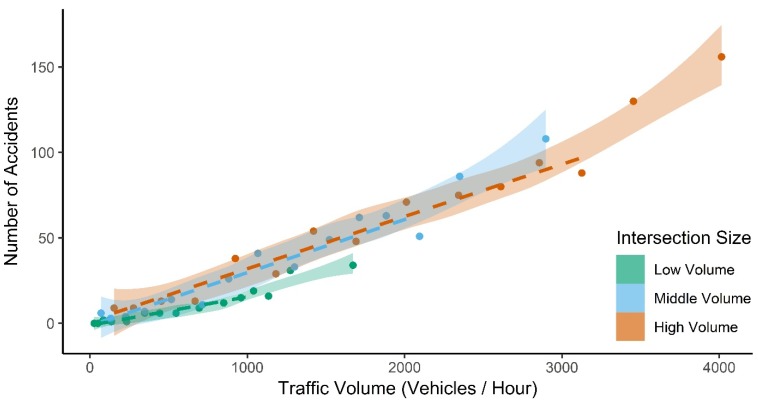
Relationship between traffic volume (median of each congestion level) and accident frequency. The dashed lines are linear regressions. For low-volume intersections, the linear regression is only fit for the median traffic volumes of the first 12 congestion levels. For middle- and high-volume intersections, the linear regression is fit for median volumes of the first 13 congestion levels. 95% confidence intervals relate to loess regressions fit to the same data.

Figure 3 emphasizes the linearity of the relationship up until the higher levels of congestion, with linear regressions fitting within the 95% confidence bands of the loess curves up until this point. For middle- and high-volume intersections, the relationship is linear up until median traffic volumes relating to congestion level 13. For low-volume intersections, the relationship is linear up until median traffic volumes relating to congestion level 12. This suggests that the statistically significant non-linear effect (Table 5) is largely a result of an exacerbated increase in accident frequency in highly congested conditions. Appendix C shows the loess regressions that the confidence bands in Figure 3 are based on.

### 3.2. Accident Severity

**Figure 4:**
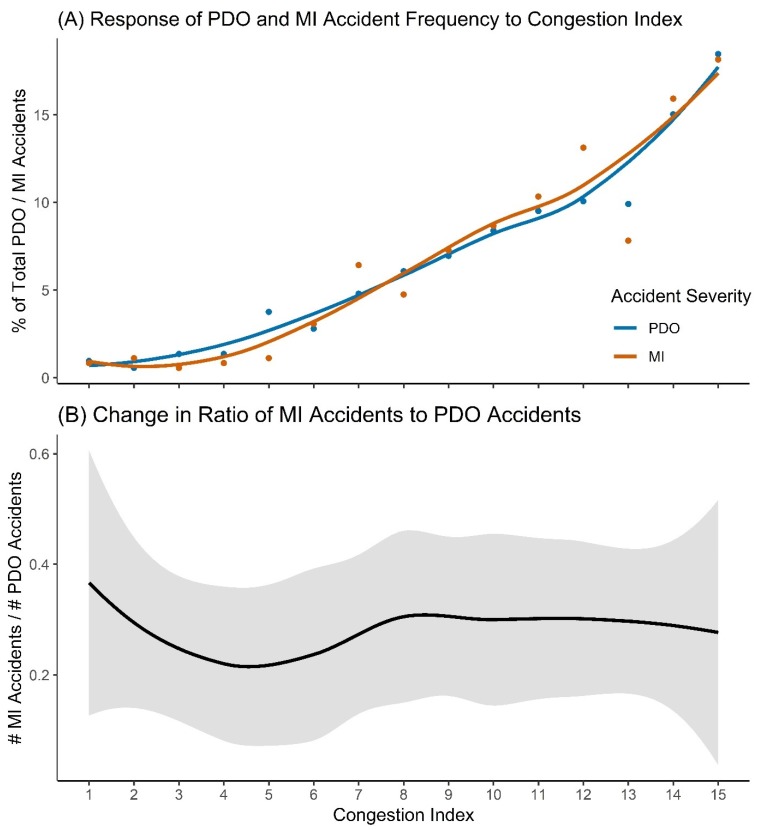
Effect of congestion on property damage only (PDO) and minor injury (MI) accidents: (**A**) Response of PDO and MI accident frequencies to congestion index. The y-axis shows normalised accident counts. The curves are loess regressions; (**B**) Change in the ratio of MI and PDO accidents with increasing congestion index level. Confidence band is 95%.

Slight differences in the response of normalised accident counts to congestion between the two severities can be seen in the loess curves (Figure 4A); however, there is likely little significance to this observation, with no change in the ratio of MI to PDO accidents being apparent (Figure 4B).

### 3.3. Rainfall Risk

**Figure 5:**
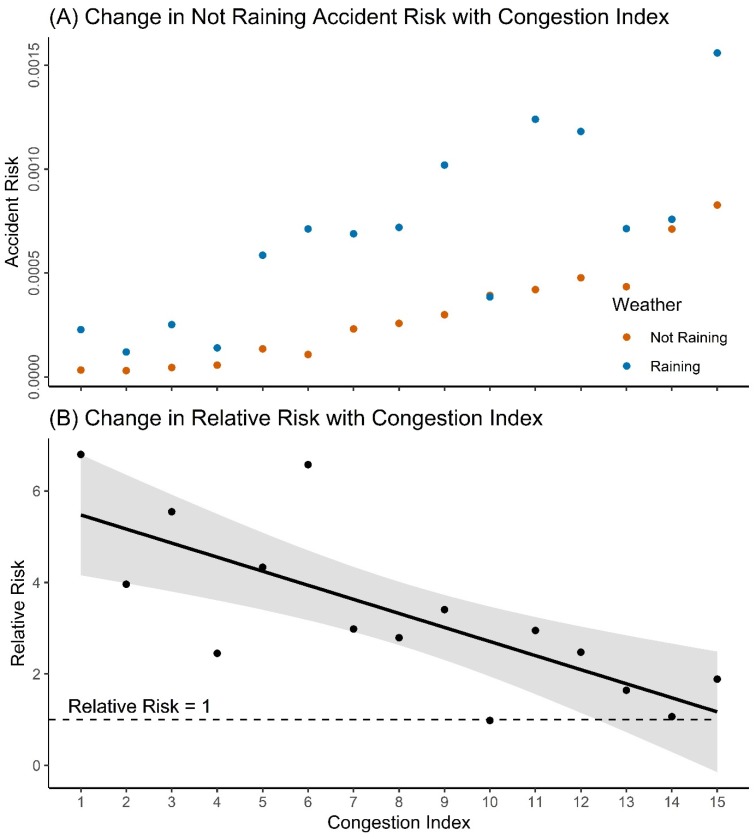
(**A**) Not-raining and raining accident risks; (**B**): Relative risk between not-raining and raining accident risks. Confidence band is 95%.

Accident risk is the probability that an accident will occur in any particular hour. Looking at congestion level 15 in Figure 5A, for example, the risk of approximately 0.0008 for not-raining accidents means that, while it is not raining, there is a 0.08% chance of an accident occurring at an intersection in the ACC in this congestion level. For both not-raining and raining accidents, the risk of an accident occurring increases with increasing congestion (Figure 5A).

**Figure A6:**
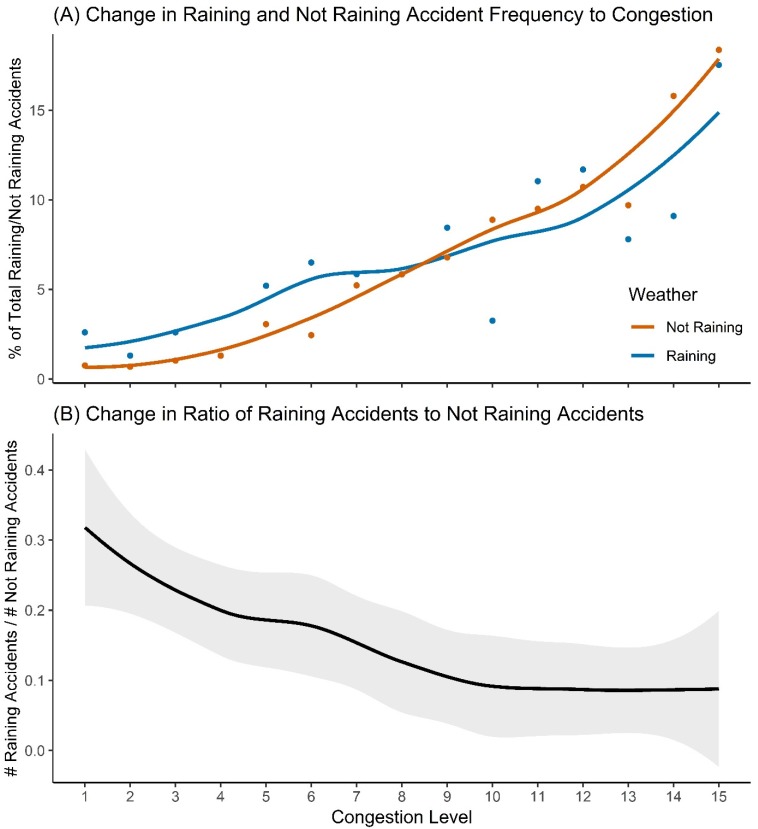
(**A**) Change in normalised not-raining and raining accident counts with increasing congestion; (**B**) Change in the ratio of raining accidents to not-raining accidents with increasing congestion. The fitted lines are loess regressions. Error bands are at a 95% confidence level.

While raining risks remain higher than not-raining risks (Figure 5A), the RR becomes smaller (Figure 5B) as congestion increases. In congestion level one, a RR of approximately five means that the risk of an accident is five times greater when it is raining than when it is not raining. By congestion level 15, the RR approaches one, which would indicate that the risk of an accident occurring while it is raining is equal to the risk of an accident occurring while it is not raining. The same conclusion can be made when looking at normalised raining and not-raining accident counts (Appendix D, Figure A6A) and the change in the ratio of raining to not-raining accident counts (Appendix D, Figure A6B).

## 4. Discussion

### 4.1. Relationship Between Traffic Volume and Accident Frequency

Results present a non-linear quadratic response between traffic volume and accident frequency in low, middle- and high-volume intersections, further increasing support for the non-linear relationship between the two variables. While the correlation is positive, as presented in most publications finding linear responses [24,25,26,27,28], the quadratic explanatory term was significant, indicating a more complex relationship. The non-linear quadratic response supports the findings of Dickerson, et al. [48] for the response of accident frequencies to traffic flow, as well as the results of Gwynn [29], Zhou and Sisiopiku [49] and Martin [31] when considering accident rates. The quadratic response emphasizes the benefit reducing congestion could have on accident occurrences. This is because reducing traffic volumes from the highest level would result in steep decline in accident occurrences compared to the same reduction at lower volumes where accident frequency increases more linearly. Comparatively, a concave relationship would indicate a reduced effectiveness of decreasing traffic volumes from the highest levels.

The ability of this analysis to detect the presence of a quadratic response may come down to several reasons.

Firstly, the quality of the traffic data used. Studies reviewed by Retallack and Ostendorf [22] using two–three million hourly traffic measurements reported concave non-linear responses. The 5.2 million data points used in this study represent a significant increase, supporting the observation that large amounts of high-quality data allow more detailed relationships to be uncovered [22]. High temporal resolution traffic data allows the precise identification of traffic conditions at the time of each accident. As accidents are rare events, obtaining large sample sizes of accidents with corresponding traffic volume data is difficult [50]. Large traffic volume datasets increase the likelihood that an accident can be paired with volume data, increasing the final sample size of accidents useable for analysis. Hossain et al. [50] note that only 30 studies in their comprehensive review had sample sizes larger than 500. Comparing this to n = 1629 in this study highlights another point where additional detail was able to be captured.

Additionally, the increased homogeneity of the highly localized study area reduces the potential of covariates to add noise to the relationship. Analysing data taken from a heterogenous study area could result in the loss of detail on a smaller scale, instead providing an averaged relationship for the area as a whole. This effect was observed by Dickerson et al. [48], where the analysis of data from four different road classes produced a linear relationship between traffic volume and accidents. When accidents were separated by road class, a non-linear effect was identified in the upper traffic volumes [48]. Similarly, Sullivan [51] was able to observe the specific effects of queuing on accident occurrence when using disaggregated data, while aggregated data only uncovered a potential effect of congestion on accident occurrence.

Other variables such as vehicle speeds and speed variation may also be considered to influence accident occurrence. However, this may be less relevant when looking at congested conditions in intersections. In non-congested conditions on straight road segments, the speeds of vehicles would have a greater influence on the risk of an accident occurring compared to congested conditions in intersections where traffic is not free-flowing and it is the number of vehicles present which has the greater effect. Although we have estimated congestion based on traffic volumes prior to accidents, secondary accidents may occur. This scenario may be addressed with more involved statistical methods using more covariates. 

We find the highest rate of increase in accident frequencies at the highest congestion levels. Hence, targeting intersections that regularly reach the highest levels of congestion may achieve the best results in terms of traffic accident management. This coincides with general benefit of reducing congestion to increase productivity and reduce delays, pollution, and stress [52].

### 4.2. Accident Severity

The lack of a clear difference of how congestion effects the occurrence of PDO or MI accidents differs from the results of Mussone, et al. [53], who found traffic volumes to be able to predict the severity of accidents. Similarly, Abdel-Aty and Keller [54] found the ADT of roads entering intersections to be significant in predicting no-injury, possible injury and incapacitating injury accidents. The lack of difference in the relationship between MI and PDO accidents may come from the low maximum vehicle speeds in the study area; with almost all intersection sites having speed limits of 50 km/h. The protection provided by the vehicle may buffer any potential influence of congestion at these relatively low speeds. If accidents including vulnerable unit types such as pedestrians and cyclists were not excluded, a difference in accident severity between congestion levels could have been found, as these unit types are more susceptible to injury.

Wang et al. [21] found congestion to have a significant influence on the occurrence of fatal and SI accidents but not MI accidents. This could indicate another reason for the lack of effect of congestion on accident severity; SI and fatal accidents were excluded due to limited sample sizes (n = 20 and n = 0, respectively).

### 4.3. Rainfall Risk

Many past studies looking at the effects of rainfall on accident risk use the matched-pair method for calculating relative risk [9,55,56,57,58,59,60]. In this approach, time periods are paired in a way that one of the periods is wet, and the other is dry. Periods are from the same time of day one week apart. The “relative risk” is then considered to be the total number of accidents in the wet periods, divided by the number of accidents in dry periods. This differs from the method used in this study, where the RR is the ratio of two proportions, rather than the ratio of raining and not-raining accident counts. The matched-pair method would not be possible for calculating RRs across the 15 congestion index levels established in our analysis. As levels of congestion are continually changing, it would be extremely difficult to find pairs of wet and dry time periods from one week to the next that also have the same congestion level.

Results suggest that in higher congestion levels the congestion itself is a more significant contributing factor to accident occurrence; with rainfall having less of an effect. This could provide insight for improved interpretation of the RR results in past studies. Hambly et al. [58], for example, investigated the wider Vancouver, Canada, region, finding a RR of 1.22. It is possible that this value is not indicative of risks in sections of the study area where high levels of congestion are common. Our results suggest that this value may be an overestimate in congested locations.

## 5. Conclusions

This study has demonstrated the ability of high temporal frequency traffic volume data to be used in parsimonious models for predicting accident frequencies at intersections. A total of 1629 motor vehicle accidents were linked traffic volume data from a pool of over 5 million hourly traffic volume data points.

Results show that accident frequency increases non-linearly in the higher levels of congestion. Therefore, suggesting that managing traffic to avoid such high levels of congestion would have the greatest impact on reducing accident occurrence. Importantly, there is no observable increase in accident frequency as congestion decreases, meaning that reducing congestion would not negatively impact public health.

Change in the severity of accidents between congestion levels was also considered. However, no relationship was found, possibly due to the lack of SI and fatal accidents in the data.

Rainfall risks were compared individually for each of the 15 levels of congestion, showing an increased influence of rainfall on accident occurrence when levels of congestion are low and indicating an increased importance of rainfall risk management in these conditions. 

This analysis has demonstrated the benefit of using long-term, broad-scale, temporally detailed data for accident risk analysis.

## Figures and Tables

**Table 1:** Accident data characteristics.

	Raw	Processed
Spatial Extent	South Australia	ACC
Temporal Extent	2010–2017	2010–2014
n	146,718	2336

**Table 2:** Intersection traffic volumes data characteristic.

	Raw	Processed
Spatial extent	ACC intersections	
Temporal extent	2010–2014	
Temporal resolution	60 minutes	
Measurement resolution	1 vehicle	
Number of intersections	122	120
n	5,369,323	5,213,580

**Table 3:** Characteristics of the created accident volumes dataset.

Accident volumes	
Spatial extent	ACC intersections
Temporal extent	2010–2014
n	1629

**Table 4:** Dispersion statistics for poisson models fitted to data for low, middle- and high-volume intersections. Dispersion ratios above one indicate possible overdispersion of data. A *p*-value of less than 0.05 indicates that the data is overdispersed.

Intersection Rank	Dispersion Ratio	Pearson’s Chi^2^	*p*-Value	Overdispersed
Low-volume	1.37	17.82	0.164	No
Middle-volume	3.77	48.99	<0.001	Yes
High-volume	3.25	42.25	<0.001	Yes

**Table 5:** Model selection statistics for models fit to accident data for low, middle- and high-volume intersections.

Model	d.f.	Log Lik.	AICc	Delta AICc	Weight	Evidence Ratio
**Low-volume intersections**						
Quadratic	3	−30.57	69.3	-	0.938	17.7
Natural spline	5	−29.21	75.1	5.8	0.053	-
Linear	2	−36.82	78.6	9.3	0.009	-
**Middle-volume intersections**						
Quadratic	4	−48.05	108.1	-	0.912	11.5
Natural spline	6	−45.25	113.0	4.9	0.079	-
Linear	3	−54.57	117.3	9.2	0.009	-
**High-volume intersections**						
Quadratic	4	−53.55	119.1	-	0.634	1.80
Natural spline	6	−48.89	120.3	1.2	0.352	-
Linear	3	−59.34	126.9	7.8	0.013	-

d.f. (degrees of freedom); AICc (Akaike Information Criterion).

**Figure A1:**
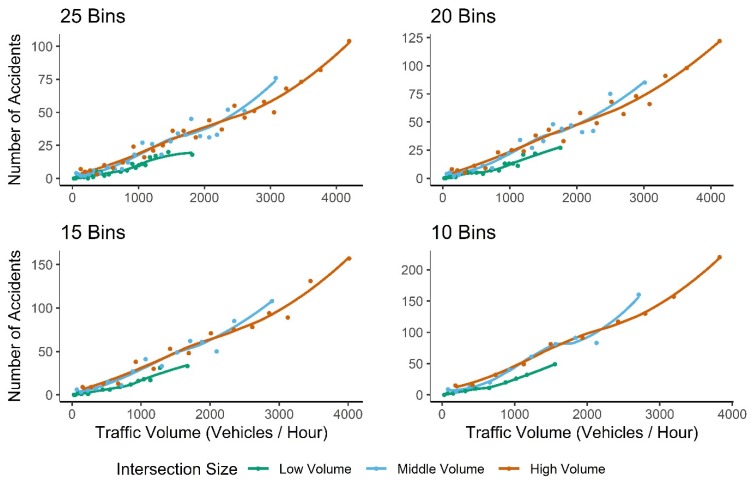
Comparison of traffic volumes grouped into 10, 15, 20 and 25 bins. Accident frequencies are plotted against the median traffic volume of each bin, and separate loess curves are fit for accidents at low, middle- and high-volume intersections.

**Figure A2:**
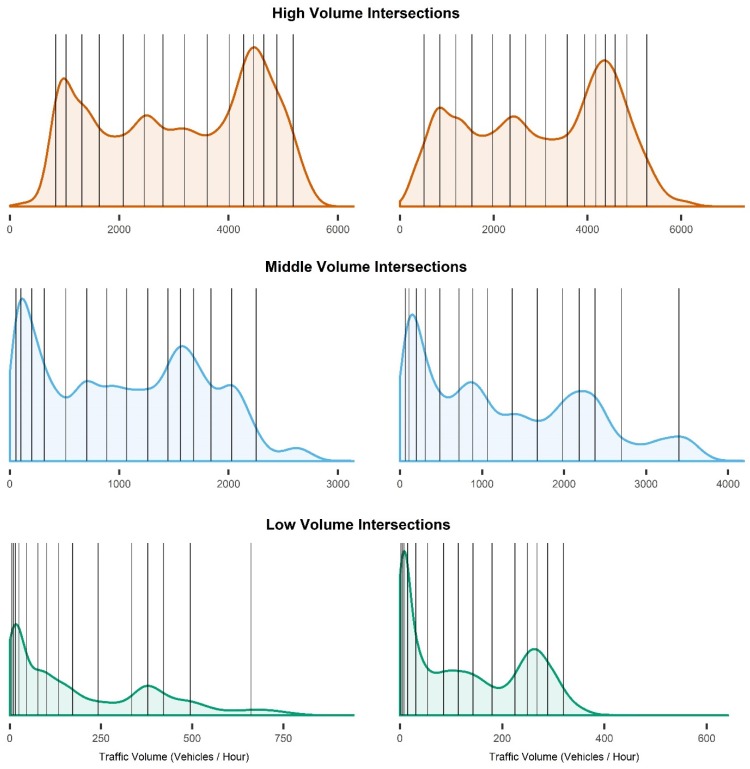
Traffic volume density distributions of the top, middle and bottom two intersection sites, ordered by median traffic volume. The lines represent where the 15 bins sit in the distribution.

**Figure A3:**
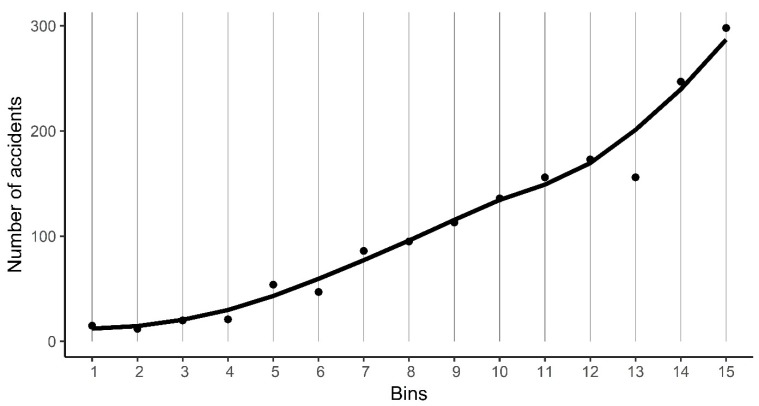
Accident frequencies plotted against the 15 traffic volume bins.

**Figure A4:**
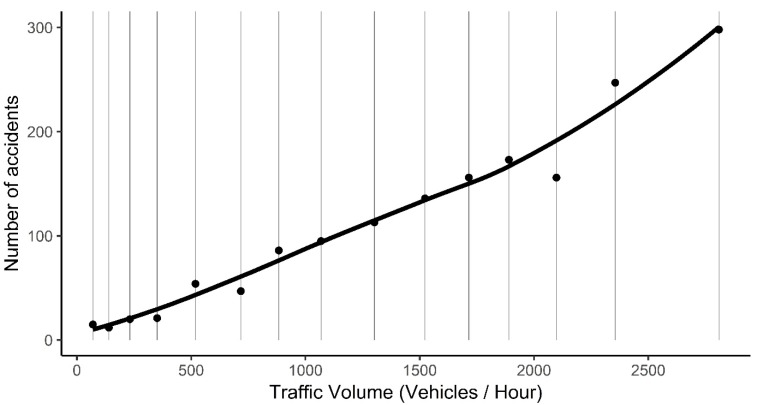
Accident frequencies plotted against the median volumes of the 15 bins.

**Figure A5:**
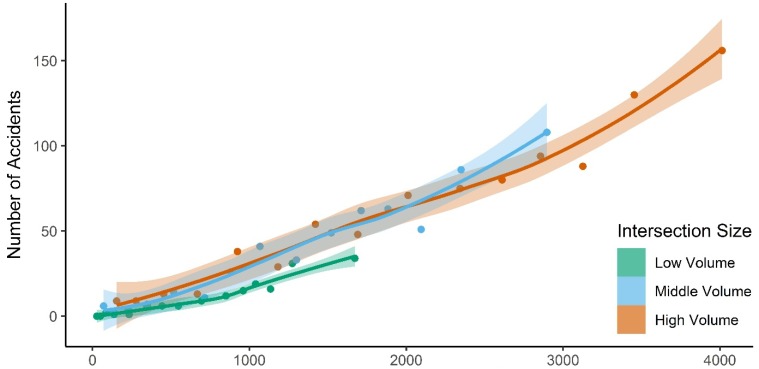
Loess curves showing the response of accident frequency to changing congestion in different sized intersections. Error bands are at a 95% confidence level.

## Appendix A. Determining the Ideal Number of Traffic Volume Bins

The Freedman–Diaconis rule [61] was used to assist in determining the ideal number of bins to divide traffic volumes into. This rule specifies that the ideal bin width is given by Equation (A1):

(A1) $$Ideal\;bin\;width=2\times \left(\frac{IQR\left(x\right)}{\sqrt[{3}]{n}}\right)$$

where x is the traffic volumes of each of the 1629 accidents and n is the number of traffic accidents.

Because accident frequencies were plotted separately for each of the three intersection traffic volume ranks, the ideal bin width was calculated for each of these groups separately. Using accidents in low-volume intersections for example:

$$Ideal\;bin\;width=2\times \left(\frac{762.25\;}{\sqrt[{3}]{158}}\right)\approx 282$$

So, traffic volumes for accidents occurring in low-volume intersections should ideally be divided into bins with widths of approximately 282 vehicles per hour. The ideal number of bins for this distribution was then calculated by dividing the total range of traffic volumes of crashes at low-volume intersections by the ideal bin width (Equation (A2)):

(A2) $$Bins=\frac{maximum\;traffic\;volume-minimum\;traffic\;volume}{ideal\;bin\;width}\approx 9.71$$

This process was repeated for accidents occurring at middle- and high-volume intersections. The results are shown in Table A1.

**Table A1:** The ideal number of bins for accidents in low, middle- and high-volume intersections calculated using the Freedman–Diaconis rule.

Intersection Rank	Ideal Bin Width	Ideal number of Bins
Low-volume	281.995	9.71
Middle-volume	299.131	16.69
High-volume	426.830	17.62
All	320.916	23.46

Based on these results, traffic volumes were divided into 10, 15, 20 and 25 bins.

However, for ease of understanding, the number of bins should remain constant throughout the report. To decide which number to choose, accident frequencies were plotted against the median traffic volumes of each of these bins, as explained in Appendix B.

Figure A1 shows the fit of loess regressions to each of the intersection volume ranks when traffic volumes are divided into 10, 15, 20 and 25 bins.

While the curve for accidents in low-volume intersections fits well when 10 bins are used, the curve for middle-volume intersections appears to be overly affected by noise. When volumes are divided into 25 bins, four of the lowest traffic volume bins contain zero accidents; using fewer bins reduces the chances of bins containing no accidents. Comparing the use of 15 and 20 bins there is little difference to the fit of the loess visually, and so 15 bins will be used as this is closer to the ideal number of bins for accidents in low-volume intersections (Table A1).

## Appendix B. Calculating the Median Traffic Volumes of Congestion Levels

Figure A2 shows how the 15 congestion levels relate to the traffic volume distributions of the highest, middle and lowest median traffic volume intersections. At the two highest volume intersections, the bins are distributed relatively evenly across the range of traffic volumes; however, in lower volume intersections the distribution becomes positively skewed. This is because high traffic volumes are rare, and as each bin contains the same number of traffic volume measurements, the bin width increases. This uneven spread of bins causes an issue when investigating the response between traffic volume and accident frequency. If accident frequencies were plotted against the 15 congestion levels on a linear scale, despite the non-linear spread seen in Figure A2, the upper end of the scale is compressed. This exaggerates the increase in accident frequency (Figure A3).

When the same accident frequency values are plotted against the median traffic volumes of the 15 bins Figure A4), the distribution of data points better represents the distribution of bins shown in Figure A2, and the response of accident frequencies is no longer affected.

However, it is important to note that taking the median traffic volumes of each of the 15 congestion index levels from all intersections will cause problems. This is due to the differences in the distribution of traffic volumes at each intersection. Consider if the median traffic volume of congestion level 15 is taken from all intersections. This volume will likely sit well below the maximum volume of a high-volume intersection and above the maximum seen at a low-volume intersection. To address this, intersections are divided into three ranks based on their median traffic volumes. The median traffic volumes of each congestion level can then be calculated separately from intersections of each rank.
